# Continuous Use During Disuse: Mechanisms and Effects of Spontaneous Activity of Unloaded Postural Muscle

**DOI:** 10.3390/ijms252212462

**Published:** 2024-11-20

**Authors:** Boris S. Shenkman, Vitaliy E. Kalashnikov, Kristina A. Sharlo, Olga V. Turtikova, Roman O. Bokov, Timur M. Mirzoev

**Affiliations:** Myology Lab, Institute of Biomedical Problems of the Russian Academy of Sciences, 123007 Moscow, Russia; vitaliy.kalasxnikov@yandex.ru (V.E.K.); sharlokris@gmail.com (K.A.S.); olga_tur@list.ru (O.V.T.); romanbokov94@yandex.ru (R.O.B.)

**Keywords:** mechanical unloading, soleus muscle, spontaneous activity, spinal motoneurons, KCC2, CLP290, intracellular signaling

## Abstract

In most mammals, postural soleus muscles are involved in the maintenance of the stability of the body in the gravitational field of Earth. It is well established that immediately after a laboratory rat is exposed to conditions of weightlessness (parabolic flight) or simulated microgravity (hindlimb suspension/unloading), a sharp decrease in soleus muscle electrical activity occurs. However, starting from the 3rd day of mechanical unloading, soleus muscle electrical activity begins to increase and reaches baseline levels approximately by the 14th day of hindlimb suspension. This phenomenon, observed in the course of rat hindlimb suspension, was named the “spontaneous electrical activity of postural muscle”. The present review discusses spinal mechanisms underlying the development of such spontaneous activity of rat soleus muscle and the effect of this activity on intracellular signaling in rat soleus muscle during mechanical unloading.

## 1. Introduction

One of the key postural muscles providing stability to the body in the Earth’s gravitational field in most mammals is the soleus muscle. The key role of the soleus muscle in maintaining a stable posture of an animal implies its prolonged daily activity (at least 11 h a day) [1]. This is possible due to the fact that 70 to 90% of muscle fibers in the soleus of humans, rats, and most mammals are slow-type fibers [2,3].

The mechanical unloading of rat hindlimbs during the first day of hindlimb suspension (HS) leads to the redistribution of contractile activity between the extensor and flexor muscles of the ankle joint and an almost complete cessation of electromyographic (EMG) activity in the soleus muscle [4]. In several laboratories, a profound decrease in electrical activity in the postural extensor muscles (such as the soleus muscle) was registered immediately after the animal’s feet were lifted off the ground. This phenomenon was observed during parabolic flights in the quadriceps muscle of dogs [5] and soleus muscle of rats [6]. A significant decline in the electrical (EMG) activity of the soleus muscle was recorded immediately after lifting the rat hindlimbs off the floor using a hindlimb suspension/unloading model and special electrodes implanted in the muscle [7,8,9]. This severely suppressed the EMG activity in the soleus muscle and lasted throughout the first 2–3 days of HS [7]. This duration of almost complete “rest” of the soleus muscle is not typical for the normal functioning of the postural muscle [1].

In the present review, we will discuss spinal mechanisms underlying the development of the soleus muscle’s spontaneous activity during unloading and outline the effect of that spontaneous activity on intracellular signaling in rat soleus muscle.

## 2. Spontaneous Activity of the Postural Soleus Muscle

**Figure 1:**
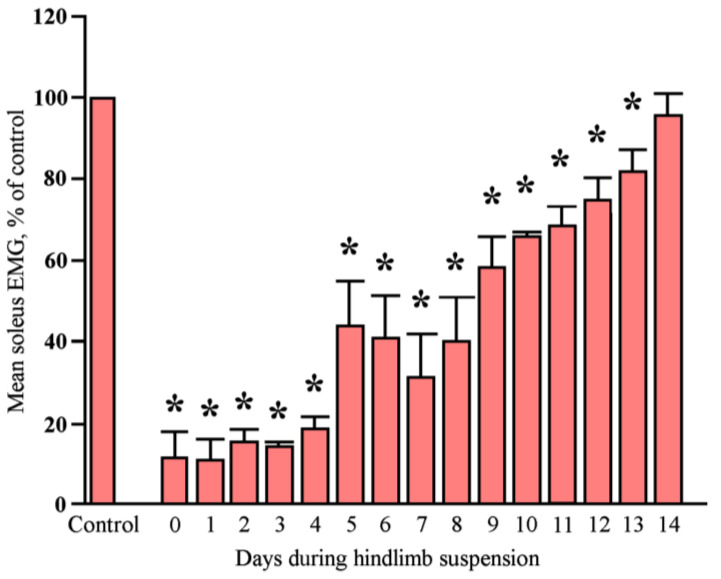
Alterations in the levels of integrated rat soleus EMG activity before and during 2-week HS. Values are means ± SE. *—significantly different from Control (pre-suspension) level (*p* < 0.05). Modified from [8].

At the end of the last century (1987), Alford and co-authors first reported that rat soleus muscle EMG activity is almost completely absent during the first 2–3 days of HS [7]. However, by the 3rd day of HS, soleus muscle resumes its electrical activity, reaching baseline levels (weight-bearing control animals) by the 14th day of HS [7]. This phenomenon can be referred to as “spontaneous postural muscle activity”. In subsequent studies [8,9], an increase in soleus muscle EMG activity under conditions of long-term HS has been repeatedly observed (Figure 1); however, the phenomenon of spontaneous activity itself and the underlying molecular mechanisms remained unexplored.

Apparently, it can be assumed that this kind of activity compensates for the sudden deficit in the contractile activity of the soleus muscle. Moreover, as will be shown below, imposing voluntary or electrically induced contractile activity can inhibit the mechanisms triggering spontaneous activity.

In 2006, Ohira et al. made an attempt to assess the functional significance of spontaneous muscle activity by applying denervation to slow (soleus) and fast (plantaris) muscles in rats during HS [10]. Denervation did not lead to a deepening of the HS-induced soleus muscle atrophy [10], which allowed us to make a cautious conclusion that spontaneous activity of the unloaded soleus muscle may not be compensatory in relation to atrophic processes, at least at the initial stage of unloading. The lack of influence of spontaneous activity on the development of atrophy may be due to the continuous nature of this activity. Indeed, continuous low-frequency rat soleus electrostimulation did not affect the size of muscle fibers under mechanical unloading [11].

**Figure 2:**
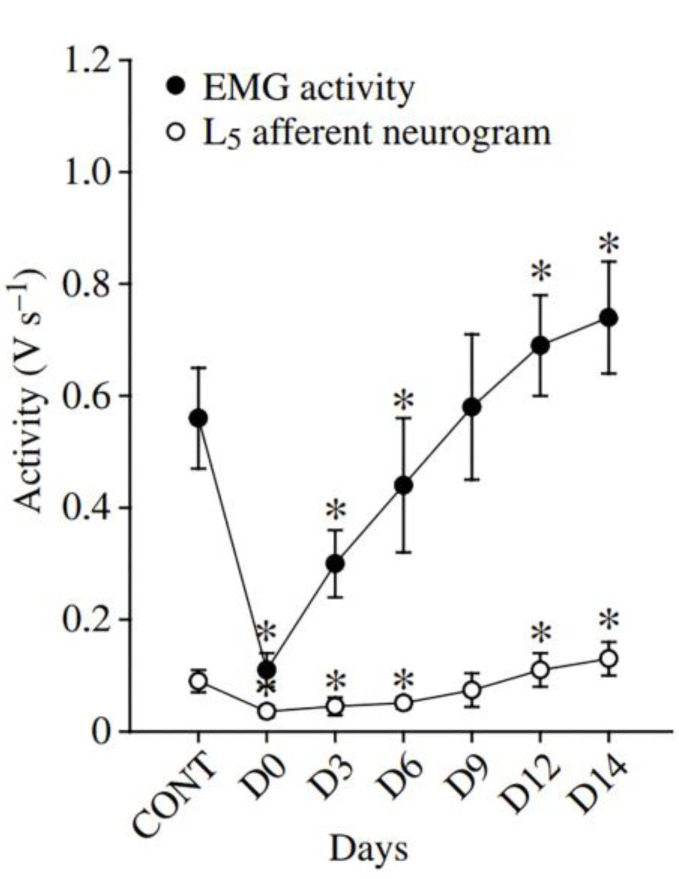
Changes in rat soleus EMG activity and L5 afferent activity (V s^–1^) during 14-day HS. Data are expressed as mean ± SD. *—significant difference from the pre-suspension (CONT) values (*p* < 0.05). Modified from [9].

In this context, the influence of afferent neuron activation on the soleus muscle EMG activity during prolonged HS is of particular interest. One study demonstrated that a profound reduction in the soleus EMG activity immediately after the onset of HS was accompanied by a decrease in the L5 afferent neurogram [9] (Figure 2). Until the 6th day of HS, EMG and afferent activities of the soleus were lower than those before HS, but they recovered between the 6th and 9th days of HS [9]. Soleus muscle EMG and afferent activities even exceeded the baseline (control) levels after 14 days of HS. Moreover, on the 1st day of recovery from HS, there was a significant boost in the EMG and afferent activities compared to those on the 14th HS day, and they regained their pre-HS levels during the 3rd and 6th recovery days [9].

These data suggest that HS cannot be regarded as a complete functional deafferentation, and in this context, it is more correct to speak only of a decrease in afferent information at the beginning of the unloading. It is possible that an increase in the afferent neurogram in parallel with an increase in spontaneous soleus muscle activity is a consequence of activation of 1a proprioceptors as a result of the increased mechanical activity of the muscle.

It is important to note that in the above studies, the soleus muscle EMG activity in rats was recorded using intramuscular electrodes. As for human studies, the registration of the surface EMG activity under unloading conditions (such as “dry” immersion), as a rule, showed a significant decrease in the basal EMG activity of the soleus muscle (usually by 30–40%), while no rise in the EMG activity was observed, at least during the first 5–7 days of “dry” immersion [12]. Apparently, a decrease in postural muscle tonic activity during unloading in humans is less pronounced than in rodents, despite the high similarity in structural, metabolic, signaling, and contractile changes with dry immersion in humans and HS in rats.

One of the manifestations of motoneuron hyperexcitability is the phenomenon of hyperreflexia. It is known that the exposure of humans to dry immersion leads to a decrease in the threshold of the H-reflex [13]. Since hyperreflexia in various pathological conditions may be associated with a decrease in the KCC2 content in the spinal cord [14], it is quite natural to assume that a decrease in the KCC2 content in spinal motoneurons and the development of spontaneous muscle activity should also occur in humans after exposure to simulated microgravity. The verification of this assumption should be the subject of future studies.

To some extent, the differences in changes in the muscle EMG activity in response to unloading may be explained by the difference in the methods of the registration of EMG activity. The registration of the electrical activity of muscles in humans is usually performed by means of percutaneous electrodes, which does not allow us to be fully confident in the precise fixation of the characteristics of the electric field of the muscle of interest, especially the soleus muscle, which lies below the gastrocnemius.

While studying the phenomenon of spontaneous postural muscle activity and its mechanisms, we found a similar phenomenon observed under conditions of spinal cord injury. The next section of the review will shed light on this issue.

## 3. Mechanisms of Increased Neuromuscular Activity and Development of Delayed-Onset Spasticity with Spinal Cord Injury

It is known that neuromuscular changes caused by the surgical isolation of the spinal cord (which eliminates supraspinal and, to a certain extent, afferent activation of motoneurons and associated muscles) are very similar to those that are observed in real or simulated microgravity (HS) [15]. The similarity of the signaling processes that cause functional reorganization of tonic/postural muscles under different types of gravitational unloading draws our attention to the phenomenon of increased excitability of motoneurons and the resulting muscle spasticity with spinal injuries.

In a number of cases, spinal cord injury leads to involuntary muscle contractions and the development of muscle spasticity localized below the site of injury [16]. Spasticity is characterized by increased muscle tone resulting from the increased excitability of motoneurons and increased synaptic inputs in response to the inactivation of inhibitory mechanisms [16].

The mechanism leading to the development of muscle spasticity with spinal cord injury was first described by Boulenguez et al. (2010) [17]. It was demonstrated that spinal cord injury-induced muscle spasticity may be caused by changes in the expression of potassium–chloride co-transporter 2 (KCC2) and sodium–potassium–chloride co-transporter (NKCC1) in motoneurons of the spinal cord [17].

**Figure 3:**
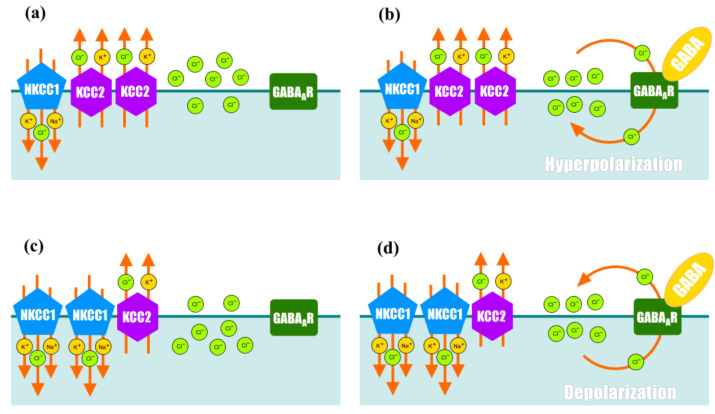
Regulation of the content of intracellular chloride ions in mature neurons and its effect on the function of GABAA receptor. (**a**) In intact mature motoneurons, KCC2 activity is higher than NKCC1 activity, resulting in a gradual outflow of chloride ions from the cytoplasm and an increase in concentration of Cl^−^ ions on the outer side of the membrane; (**b**) binding of GABA to its receptors on the membrane surface opens ion channels, resulting in an influx of Cl^−^ ions into the motoneuron and subsequent membrane hyperpolarization; (**c**) in immature motoneurons and after spinal cord injury, higher levels of NKCC1 and lower levels of KCC2 lead to the accumulation of Cl^−^ ions at the inner side of the membrane; (**d**) the opening of ion channels caused by binding of GABA to its receptors causes outflow of Cl^−^ ions and membrane depolarization.

It is well known that in immature neurons, NKCC1 is actively expressed, whereas the expression of KCC2 is downregulated. High NKCC1 activity leads to the accumulation of chloride ions within the neuron [18]. The binding of GABA to its receptor triggers an ionic current through chloride channels, and chloride ions, in accordance with a concentration gradient, leave the cell, leading to the depolarization of the membrane and the development of an action potential [18] (Figure 3).

In mature neurons, the expression and activity of NKCC1 is low, whereas KCC2 is highly expressed [18]. This leads to a significant decrease in the intracellular concentration of chloride ions (<10 mmol/L) [18]. In this case, the binding of GABA to its receptor leads to the opening of ion channels on the membrane and the movement of chloride ions into the cytoplasm with the subsequent hyperpolarization of the membrane [18] (Figure 3). Membrane hyperpolarization suppresses action potentials acting on the motoneurons by increasing the stimulus required to shift the membrane potential to reach the action potential threshold [18]. Thus, GABA serves as the major inhibitory neurotransmitter in mature neurons.

**Figure 4:**
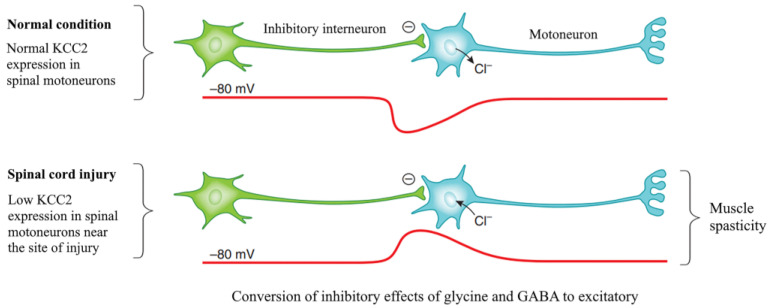
Spinal cord injury may result in reduced expression of KCC2 in motoneuron membranes resulting in a more positive equilibrium potential for chloride ions, a switch in synaptic input from inhibitory to excitatory, motoneuron hyperactivity, and muscle spasticity. Modified from [16].

It has been demonstrated that spinal cord injury reproduces some characteristics of immature motoneurons, namely, the content of KCC2 and the outflow of chloride ions is downregulated, while the content of NKCC1 and the influx of chloride ions is increased [17]. This leads to a positive shift in the resting potential (from −75 to −65 mV), replacing the inhibitory effects of glycine and GABA with excitatory ones, and causes a spontaneous development of the action potential, an increase in muscle activity, and, subsequently, muscle spasticity [16] (Figure 4). This mechanism has been found not only in humans and animals with spinal cord injuries but also in cases of cerebral circulatory disorders and in models of cerebral palsy [19,20]. Accordingly, researchers are looking for the cause of such changes in chloride homeostasis and subsequent events in spinal motoneurons. In particular, researchers are interested in the mechanisms implicated in the reduction in KCC2 in neurons.

Recently, it has been shown that the inactivation of the calpain-1 (a well-known calcium-dependent cysteine protease) in animals with spinal cord injury prevents a decrease in KCC2 content and subsequent muscle spasticity [21]. The results of this study suggest that it is calpain-dependent proteolysis that is the main cause of KCC2 reduction in the lumbar spinal cord. At the same time, there is evidence that the reduced KCC2 protein content in spinal motoneurons can be attributed to decreased KCC2 mRNA expression [22]. What events are responsible for triggering these mechanisms? A decrease in KCC2 content was also found in axotomized spinal cord motoneurons [22]. These data suggest that such KCC2 downregulation may indicate the contribution of neuromuscular activity to the regulation of KCC2 content in motoneurons.

Interestingly, it has been recently shown that voluntary physical exercises in animal models of spinal cord injury and stroke can prevent a decrease in the KCC2 content in spinal motoneurons [23,24]. In particular, Li et al. (2022) demonstrated that body weight-supported treadmill training ameliorates motoneuronal hyperexcitability in rats with incomplete spinal cord injury by increasing KCC2 expression in spinal motoneurons [24]. However, when animals with spinal cord injury were injected with antibodies against Trkb receptors (receptors for brain-derived neurotrophic factor, BDNF), the effect of treadmill training was completely eliminated [24]. At the same time, KCC2 content in spinal motoneurons decreased suggesting the maintenance of spontaneous muscle activity and spasticity [24].

Thus, the activity of the neuromuscular apparatus can be considered one of the main regulators of the KCC2 content in spinal motoneurons. When motoneurons are “switched off” in the case of spinal cord injuries, impaired cerebral circulation, and other pathological changes, a decrease in KCC2 content is observed with a subsequent change in the chloride homeostasis of the cell and a significant increase in its excitability. As a result of these changes, motoneurons generate spontaneous muscle activity, resulting in muscle spasticity. However, if the motor activity is imposed on an animal under the impaired central regulation of motoneurons, no changes in KCC2 content, chloride homeostasis, and excitability occur. It is possible that BDNF, which is secreted by a contracting muscle, can serve as a mediator that signals to motoneurons about the active state of muscles.

The key role of the KCC2/NKCC1 balance in the pathogenesis of various neurological disorders, including refractory epilepsy, neuropathic pain [25] and muscle spasticity due to spinal cord injury [17], makes these co-transporters important therapeutic targets for the treatment of neurological diseases. All these circumstances contribute to the accumulation of data on effective methods of pharmacological modulation of KCC2 and NKCC1 expression. Currently, prochlorperazine [26] and CLP (CLP-257 and CLP 290) [27] have been demonstrated to increase KCC2 content in lumbar motoneurons in rats. The common feature of these drugs is the ability to enhance KCC2 expression in spinal motoneurons and thereby reduce hyperreflexia and muscle spasticity after spinal cord injury.

Thus, several pharmacological agents that regulate potassium–chloride transport by preventing a reduction in KCC2 expression/content can be used in experimental studies on spontaneous muscle activity. Therefore, prochlorperazine and CLP were used to investigate the mechanisms and effects of spontaneous muscle activity during gravitational unloading.

## 4. Spinal Mechanisms of Spontaneous Muscle Activity and Its Effects on Soleus Muscle During Gravitational Unloading

**Figure 5:**
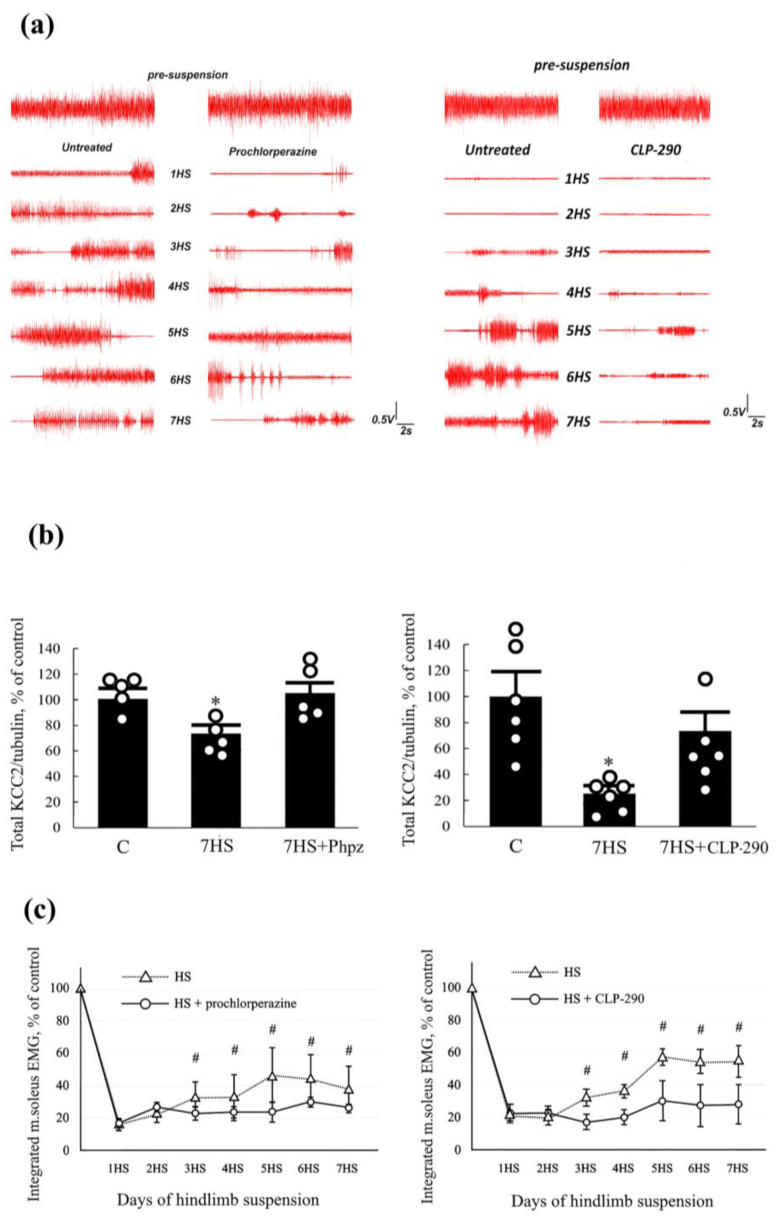
Changes in rat soleus muscle EMG activity and KCC2 content in spinal motoneurons with prochlorperazine and CLP-290 administration during hindlimb suspension (HS). (**a**) Typical patterns of EMG activity of rat soleus before and during HS with and without prochlorperazine or CLP-290 administration; (**b**) KCC2 content in rat lumbar spinal cord after administration of prochlorperazine or CLP290; (**c**) integral EMG activity of rat soleus HS with and without administration of prochlorperazine and CLP-290. C—control, 1HS—7HS—days of hindlimb suspension, 7HS + Phpz—7-day HS with prochlorperazine administration, 7 HS + CLP-290—7-day HS with CLP290 administration, *—significant difference from control (*p* < 0.05). #—significant difference from HS + Phpz or HS + CLP-290 (*p* < 0.05). Adapted from [28,29].

Similarities between spinal cord injury and hindlimb unloading in terms of time-course changes in spinal motoneurons and EMG activity of ankle extensors allowed us to formulate and experimentally test the hypothesis that the development of hyperexcitability and the spontaneous activity of spinal motoneurons during gravitational unloading is due to a decrease in KCC2 content [28] (Figure 5). Indeed, it turned out that a decrease in KCC2 content and an increase in NKCC1 content in lumbar motoneurons of rats already occurs after 7 days of HS [28]. At the same time, in accordance with previous studies, starting from the 2nd day of HS, there is a rise in the soleus EMG activity as registered by intramuscular electrodes [28] (Figure 5). Prochlorperazine administration during 7-day HS prevented a decrease in the KCC2 content in lumbar motoneurons and kept soleus muscle EMG activity at low levels during the entire period of HS [28] (Figure 5). 

Similar effects were observed with chronic CLP290 administration during 7-day HS [29] (Figure 5). Thus, it was shown that prevention of a decrease in KCC2 expression in the lumbar spinal cord by pharmacological agents of a different nature leads to an almost complete elimination of rat soleus spontaneous activity during hindlimb unloading.

In order to elucidate the role of the spontaneous muscle activity in the processes of molecular reorganization of postural muscle under unloading conditions, it is necessary to evaluate molecular changes in atrophying muscle that occur during a significant decrease (or elimination) of spontaneous activity. This can be performed using drugs that prevent an HS-induced decrease in KCC2 content. In our experiments, we analyzed the signaling responses of rat soleus muscle under the pharmacological blocking of spontaneous activity during HS. The chronic administration of prochlorperazine during HS, which can have a side effect on calcium homeostasis [30,31], resulted in suppressed soleus muscle EMG (and, accordingly, contractile) activity and paradoxical changes in the markers of several signaling pathways, including (1) the prevention of an increased expression of muscle-specific ubiquitin ligases (MuRF-1 and MAFbx/atrogin-1) during unloading and (2) the prevention of a decrease in ribosomal RNA content [32].

**Figure 6:**
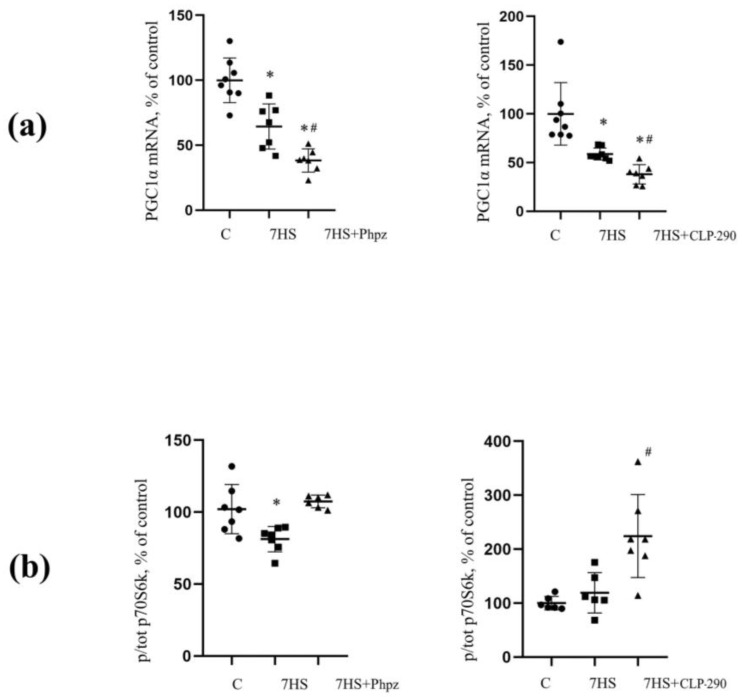
Effects of prochlorperazine and CLP-290 administration during HS on PGC1α expression and p70S6K phosphorylation in rat soleus muscle. (**a**) Changes in PGC1α mRNA expression in rat soleus muscle with prochlorperazine and CLP-290 administration during 7-day hindlimb suspension (HS); (**b**) Changes in p70S6K (Thr389) phosphorylation in rat soleus muscle with prochlorperazine and CLP-290 administration during 7-day hindlimb suspension. C—control, 7 HS—7 days of hindlimb suspension, 7 HS + Phpz—7-day HS with prochlorperazine administration, 7 HS + CLP-290—7-day HS with CLP290 administration, *—significant difference from control (*p* < 0.05), #—significant difference from 7 HS (*p* < 0.05). Adapted from [33,34].

Moreover, prochlorperazine-related suppression of the spontaneous activity reduced the atrophy of slow and fast soleus muscle fibers in unloaded rats [32]. Interestingly, chronic administration of another KCC2 activator, CLP290, had no effect on ubiquitin ligases and rRNAs in unloaded soleus muscle [33]. Furthermore, no changes in the size of slow and fast soleus muscle fibers were detected. At the same time, both prochlorperazine and CLP290 induced a more profound decrease in PGC1α mRNA expression (a key regulator of mitochondrial biogenesis) and some enzymes of oxidative phosphorylation compared to HS rats without prochlorperazine or CLP290 treatment [33,34] (Figure 6). This fact confirms the notion that the functional state of mitochondria appears to be in correlation with the level of muscle contractile activity [35]. It can be assumed that the emergence of the spontaneous soleus muscle activity during unloading can partly prevent an HS-induced decrease in the markers of mitochondrial biogenesis.

It is important to note that the phosphorylation levels of ribosomal protein S6 kinase (p70S6K) (a downstream target of the mechanistic target of rapamycin complex 1, mTORC1) were significantly increased in unloaded animals with the CLP290-related suppression of spontaneous soleus muscle activity [33] (Figure 6). At the same time, in unloaded rats without CLP290 treatment and continuous spontaneous soleus muscle activity, p70S6K phosphorylation did not differ from the control group (Figure 6).

Previously, an increase in p70S6K phosphorylation has been shown in rat soleus muscle after 6 and 24 h of HS [36,37]. The increased phosphorylation of p70S6K during the early stages of disuse has also been demonstrated with muscle immobilization [38] and denervation [39]. Such an increase in p70S6K phosphorylation is probably related to the transition of the soleus muscle from continuous activity to almost complete inactivity (for review, see [40]). During this transition, the suppression of anabolic processes slows down, and anabolic signaling pathways in muscle fibers are activated [41]. These changes are carried out via a number of mechanisms, the contribution of which is yet to be evaluated.

While analyzing the effects of spontaneous activity on p70S6K phosphorylation, it is important to note that the spontaneous soleus muscle activity in rats from the HS group (without CLP290) is continuous, i.e., without periods of relaxation/rest. Such an activity pattern is not typical for postural soleus muscle under normal conditions. When soleus muscle spontaneous activity is reduced with CLP290, we may expect the suppression of those molecular mechanisms that prevent the activation of anabolic signaling (p70S6K phosphorylation) under normal soleus muscle contractile activity. These processes result in the activation of p70S6K. Further studies will show to what extent our hypothetical ideas correspond to real facts.

As noted above, a reduction in muscle contractile activity by axotomy leads to a decrease in the KCC2 content in lumbar motoneurons even without disturbing spinal cord integrity [22]. In animals with incomplete transection of the spinal cord above the lumbar segment, body weight-supported treadmill training prevented a decrease in the KCC2 content in motoneurons [24]. These facts indicate that the content of KCC2 in spinal motoneurons is highly dependent on the preceding activity of the neuromuscular apparatus. In our recent studies, we have found that chronic low-frequency electrical stimulation of rat soleus during HS partly prevented a decrease in the KCC2 content in the lumbar spinal cord (unpublished observation). 

Thus, it can be assumed that the first 2–3 days of unloading conditions create prerequisites for an increase in the excitability of motoneurons and the development of spontaneous postural muscle activity. Thus, if increased contractile activity is imposed on the soleus muscle during mechanical unloading, the spontaneous activity either does not occur or is significantly reduced. It is clear that this hypothesis needs to be tested in future experiments.

## 5. Conclusions and Future Directions

The present review considers the existing literature and our own data, describing a previously poorly explored phenomenon: the spontaneous activity of postural muscle under conditions of mechanical unloading. Indeed, it turned out that after a 2–3-day period of almost complete inactivity of unloaded soleus muscle, a gradual increase in soleus EMG activity spontaneously appears, and, by the 14th day of unloading, this EMG activity reaches baseline levels. The first experiments, although not very rigorous, showed that this activity does not affect the degree of muscle atrophy and therefore cannot be considered as compensatory in the full sense of the word. However, neuronal mechanisms and the signaling role of this activity remained unexplored for a long time. Studies conducted in our lab have shown that under unloading conditions, as in the case of spinal cord injury, the spontaneous activity of the soleus muscle is accompanied by a decrease in the content of KCC2 in the lumbar spinal cord and a subsequent increase in the excitability of motoneurons. At the same time, the pharmacological prevention of a decrease in KCC2 content leads to a decrease or almost complete elimination of such spontaneous muscle activity. There is also some evidence suggesting that a decrease in KCC2 and the appearance of spontaneous activity are the result of complete muscle inactivity during the first days of unloading. Thus, the first steps have been taken toward uncovering the mechanisms of spontaneous activity of the postural muscle under unloading. It was also shown that a decrease in spontaneous activity induces significant changes in a number of signaling pathways in rat soleus muscle.

At the same time, many important questions related to the mechanisms of spontaneous activity and its role in the development of atrophic processes under unloading conditions remain unanswered. First of all, there is no direct evidence of the presence of spontaneous soleus muscle activity in humans under various types of unloading. Second, we have no evidence of the compensatory role of the spontaneous activity under the ongoing atrophic process. At present, we can only talk about the fact that spontaneous activity allows for the expression of some markers of mitochondrial biogenesis above the minimal level (see above). The maintenance of muscle fiber size or markers of anabolic signaling pathways is not supported by any evidence.

Moreover, the parameters, mechanisms, and consequences of spontaneous muscle activity during longer periods of gravitational unloading have not yet been analyzed. Molecular mechanisms underlying the development of spontaneous activity during longer periods of gravitational unloading are still unclear and require further research.

## References

[1] Hodgson J.A., Roy R.R., Higuchi N., Monti R.J., Zhong H., Grossman E., Edgerton V.R. (2005). Does daily activity level determine muscle phenotype?. J. Exp. Biol..

[2] Johnson M.A., Polgar J., Weightman D., Appleton D. (1973). Data on the distribution of fibre types in thirty-six human muscles. An autopsy study. J. Neurol. Sci..

[3] Delp M.D., Duan C. (1996). Composition and size of type I, IIA, IID/X, and IIB fibers and citrate synthase activity of rat muscle. J. Appl. Physiol..

[4] Shenkman B.S., Mirzoev T.M., Kozlovskaya I.B. (2020). Tonic activity and gravitational control of postural muscle. Aerosp. Environ. Med..

[5] Yuganov E.M., Kasyan I.I., Asyamolov B.F. (1963). Bioelectrical activity of skeletal muscle under condition of intermittent action of overloading and weightlessness. Izv. Akad. Nauk. SSSR. Ser. Biolog..

[6] Kawano F., Nomura T., Ishihara A., Nonaka I., Ohira Y. (2002). Afferent input-associated reduction of muscle activity in microgravity environment. Neuroscience.

[7] Alford E.K., Roy R.R., Hodgson J.A., Edgerton V.R. (1987). Electromyography of rat soleus, medial gastrocnemius, and tibialis anterior during hind limb suspension. Exp. Neurol..

[8] Kawano F., Ishihara A., Stevens J.L., Wang X.D., Ohshima S., Horisaka M., Maeda Y., Nonaka I., Ohira Y. (2004). Tension- and afferent input-associated responses of neuromuscular system of rats to hindlimb unloading and/or tenotomy. Am. J. Physiology. Regul. Integr. Comp. Physiol..

[9] De-Doncker L., Kasri M., Picquet F., Falempin M. (2005). Physiologically adaptive changes of the L5 afferent neurogram and of the rat soleus EMG activity during 14 days of hindlimb unloading and recovery. J. Exp. Biol..

[10] Ohira Y., Yoshinaga T., Ohara M., Kawano F., Wang X.D., Higo Y., Terada M., Matsuoka Y., Roy R.R., Edgerton V.R. (2006). The role of neural and mechanical influences in maintaining normal fast and slow muscle properties. Cells Tissues Organs.

[11] Dupont E., Cieniewski-Bernard C., Bastide B., Stevens L. (2011). Electrostimulation during hindlimb unloading modulates PI3K-AKT downstream targets without preventing soleus atrophy and restores slow phenotype through ERK. Am. J. Physiol. Regul. Integr. Comp. Physiol..

[12] Grigor’ev A.I., Kozlovskaia I.B., Shenkman B.S. (2004). The role of support afferents in organization of the tonic muscle system. Ross. Fiziol. Zhurnal Im. I.M. Sechenova.

[13] Kozlovskaya I., Dmitrieva I., Grigorieva L., Kirenskaya A.V., Kreidich Y.V., Gurfinkel V.S., Ioffe M.E., Massion J. (1988). Gravitational Mechanisms in the Motor System. Studies in Real and Simulated Weightlessness. Stance and Motion: Facts and Concepts.

[14] Bilchak J.N., Yeakle K., Caron G., Malloy D., Cote M.P. (2021). Enhancing KCC2 activity decreases hyperreflexia and spasticity after chronic spinal cord injury. Exp. Neurol..

[15] Baldwin K.M., Haddad F., Pandorf C.E., Roy R.R., Edgerton V.R. (2013). Alterations in muscle mass and contractile phenotype in response to unloading models: Role of transcriptional/pretranslational mechanisms. Front. Physiol..

[16] Edgerton V.R., Roy R.R. (2010). Spasticity: A switch from inhibition to excitation. Nat. Med..

[17] Boulenguez P., Liabeuf S., Bos R., Bras H., Jean-Xavier C., Brocard C., Stil A., Darbon P., Cattaert D., Delpire E. (2010). Down-regulation of the potassium-chloride cotransporter KCC2 contributes to spasticity after spinal cord injury. Nat. Med..

[18] Koumangoye R., Bastarache L., Delpire E. (2021). NKCC1: Newly Found as a Human Disease-Causing Ion Transporter. Function.

[19] Toda T., Ishida K., Kiyama H., Yamashita T., Lee S. (2014). Down-regulation of KCC2 expression and phosphorylation in motoneurons, and increases the number of in primary afferent projections to motoneurons in mice with post-stroke spasticity. PLoS ONE.

[20] Coq J.O., Delcour M., Ogawa Y., Peyronnet J., Castets F., Turle-Lorenzo N., Montel V., Bodineau L., Cardot P., Brocard C. (2018). Mild Intrauterine Hypoperfusion Leads to Lumbar and Cortical Hyperexcitability, Spasticity, and Muscle Dysfunctions in Rats: Implications for Prematurity. Front. Neurol..

[21] Kerzonkuf M., Verneuil J., Brocard C., Dingu N., Trouplin V., Ramirez Franco J.J., Bartoli M., Brocard F., Bras H. (2024). Knockdown of calpain1 in lumbar motoneurons reduces spasticity after spinal cord injury in adult rats. Mol. Ther. J. Am. Soc. Gene Ther..

[22] Akhter E.T., Griffith R.W., English A.W., Alvarez F.J. (2019). Removal of the Potassium Chloride Co-Transporter from the Somatodendritic Membrane of Axotomized Motoneurons Is Independent of BDNF/TrkB Signaling But Is Controlled by Neuromuscular Innervation. eNeuro.

[23] Hugues N., Pin-Barre C., Brioche T., Pellegrino C., Berton E., Rivera C., Laurin J. (2023). High-intensity training with short and long intervals regulate cortical neurotrophic factors, apoptosis markers and chloride homeostasis in rats with stroke. Physiol. Behav..

[24] Li X., Song X., Fang L., Ding J., Qi L., Wang Q., Dong C., Wang S., Wu J., Wang T. (2022). Body Weight-Supported Treadmill Training Ameliorates Motoneuronal Hyperexcitability by Increasing GAD-65/67 and KCC2 Expression via TrkB Signaling in Rats with Incomplete Spinal Cord Injury. Neurochem. Res..

[25] Tang B.L. (2020). The Expanding Therapeutic Potential of Neuronal KCC2. Cells.

[26] Liabeuf S., Stuhl-Gourmand L., Gackiere F., Mancuso R., Sanchez Brualla I., Marino P., Brocard F., Vinay L. (2017). Prochlorperazine Increases KCC2 Function and Reduces Spasticity after Spinal Cord Injury. J. Neurotrauma.

[27] Gagnon M., Bergeron M.J., Lavertu G., Castonguay A., Tripathy S., Bonin R.P., Perez-Sanchez J., Boudreau D., Wang B., Dumas L. (2013). Chloride extrusion enhancers as novel therapeutics for neurological diseases. Nat. Med..

[28] Kalashnikov V.E., Tyganov S.A., Turtikova O.V., Kalashnikova E.P., Glazova M.V., Mirzoev T.M., Shenkman B.S. (2021). Prochlorperazine Withdraws the Delayed Onset Tonic Activity of Unloaded Rat Soleus Muscle: A Pilot Study. Life.

[29] Kalashnikov V.E., Sergeeva K.V., Turtikova O.V., Tyganov S.A., Mirzoev T.M., Shenkman B.S. (2024). Spontaneous Tonic Activity Revealed in Rat Soleus Muscle by CLP290, a Novel Spinal Cord Potassium-Chloride Cotransporter Activator, during Hindlimb Suspension. J. Evol. Biochem. Physiol..

[30] Richelson E., Nelson A. (1984). Antagonism by neuroleptics of neurotransmitter receptors of normal human brain In Vitro. Eur. J. Pharmacol..

[31] Belkacemi L., Darmani N.A. (2020). Dopamine receptors in emesis: Molecular mechanisms and potential therapeutic function. Pharmacol. Res..

[32] Sergeeva K.V., Sharlo K.A., Kalashnikov V.E., Turtikova O.V., Tyganov S.A., Shenkman B.S. (2023). The Effect of Spontaneous Neuromuscular Activity on the Development of Atrophy of the Functionally Unloaded m. Soleus. Ross. Fiziol. Zhurnal Im. I.M. Sechenova.

[33] Sergeeva X.V., Sharlo K.A., Tyganov S.A., Kalashnikov V.E., Shenkman B.S. (2024). Molecular Signaling Effects behind the Spontaneous Soleus Muscle Activity Induced by 7-Day Rat Hindlimb Suspension. Int. J. Mol. Sci..

[34] Sharlo K.A., Lvova I.D., Tyganov S.A., Sergeeva K.V., Kalashnikov V.Y., Kalashnikova E.P., Mirzoev T.M., Kalamkarov G.R., Shevchenko T.F., Shenkman B.S. (2023). A Prochlorperazine-Induced Decrease in Autonomous Muscle Activity during Hindlimb Unloading Is Accompanied by Preserved Slow Myosin mRNA Expression. Curr. Issues Mol. Biol..

[35] Memme J.M., Erlich A.T., Phukan G., Hood D.A. (2021). Exercise and mitochondrial health. J. Physiol..

[36] Mirzoev T., Tyganov S., Vilchinskaya N., Lomonosova Y., Shenkman B. (2016). Key Markers of mTORC1-Dependent and mTORC1-Independent Signaling Pathways Regulating Protein Synthesis in Rat Soleus Muscle During Early Stages of Hindlimb Unloading. Cell. Physiol. Biochem. Int. J. Exp. Cell. Physiol. Biochem. Pharmacol..

[37] Chibalin A.V., Benziane B., Zakyrjanova G.F., Kravtsova V.V., Krivoi I.I. (2018). Early endplate remodeling and skeletal muscle signaling events following rat hindlimb suspension. J. Cell. Physiol..

[38] You J.S., Anderson G.B., Dooley M.S., Hornberger T.A. (2015). The role of mTOR signaling in the regulation of protein synthesis and muscle mass during immobilization in mice. Dis. Models Mech..

[39] Tang H., Inoki K., Lee M., Wright E., Khuong A., Khuong A., Sugiarto S., Garner M., Paik J., DePinho R.A. (2014). mTORC1 promotes denervation-induced muscle atrophy through a mechanism involving the activation of FoxO and E3 ubiquitin ligases. Sci. Signal..

[40] Shenkman B.S. (2023). From activity to inactivity, and back to activity again. Signaling processes in the postural muscle during the transition period. Aerosp. Environ. Med..

[41] Rennie M.J. (2005). Why muscle stops building when it’s working. J. Physiol..

